# Changes in Intracellular Na^+^ following Enhancement of Late Na^+^ Current in Virtual Human Ventricular Myocytes

**DOI:** 10.1371/journal.pone.0167060

**Published:** 2016-11-22

**Authors:** Karen Cardona, Beatriz Trenor, Wayne R. Giles

**Affiliations:** 1 Centro de Investigación e Innovación en Bioingeniería, Universitat Politècnica de València, Valencia, Spain; 2 Faculty of Kinesiology, University of Calgary, Calgary, Alberta, Canada; The Ohio State University, UNITED STATES

## Abstract

The slowly inactivating or late Na^+^ current, I_Na-L_, can contribute to the initiation of both atrial and ventricular rhythm disturbances in the human heart. However, the cellular and molecular mechanisms that underlie these pro-arrhythmic influences are not fully understood. At present, the major working hypothesis is that the Na^+^ influx corresponding to I_Na-L_ significantly increases intracellular Na^+^, [Na^+^]_i_; and the resulting reduction in the electrochemical driving force for Na^+^ reduces and (may reverse) Na^+^/Ca^2+^ exchange. These changes increase intracellular Ca^2+^, [Ca^2+^]_i_; which may further enhance I_Na-L_ due to calmodulin-dependent phosphorylation of the Na^+^ channels. This paper is based on mathematical simulations using the O’Hara et al (2011) model of baseline or healthy human ventricular action potential waveforms(s) and its [Ca^2+^]_i_ homeostasis mechanisms. Somewhat surprisingly, our results reveal only very small changes (≤ 1.5 mM) in [Na^+^]_i_ even when I_Na-L_ is increased 5-fold and steady-state stimulation rate is approximately 2 times the normal human heart rate (i.e. 2 Hz). Previous work done using well-established models of the rabbit and human ventricular action potential in heart failure settings also reported little or no change in [Na^+^]_i_ when I_Na-L_ was increased. Based on our simulations, the major short-term effect of markedly augmenting I_Na-L_ is a significant prolongation of the action potential and an associated increase in the likelihood of reactivation of the L-type Ca^2+^ current, I_Ca-L_. Furthermore, this action potential prolongation does not contribute to [Na^+^]_i_ increase.

## Introduction

Precise tuning and homeostatic regulation of intracellular Na^+^ levels, [Na^+^]_i_, are known to be essential elements of a number of very important regulatory physiological processes in mammalian heart cells (cf. [1–3]). These include: modulation of cell volume as a consequence of the dependence of Na^+^/K^+^ pump activity on [Na^+^]_i_ [4,5]; the strong dependence of steady-state intracellular Ca^2+^ levels, [Ca^2+^]_i_, on the electrochemical gradient for Na^+^, conferred by the activity of the Na^+^/Ca^2+^ exchanger and associated electrogenic current [1,6]; and regulation of intracellular pH, due to both Na^+^/H^+^ exchange [7], and Na^+^/HCO_3_ exchange [8]. Changes in [Na^+^]_i_ can strongly regulate the contractile state of ventricular myocytes. This is especially the case for resting tension in isolated preparations (e.g. trabeculae) [9,10] or the diastolic pressure in the whole heart. The recognition that there is a significant Na^+^ influx into cardiac myocytes with each action potential has been the basis for detailed studies of the rate-dependence of [Na^+^]_i_ and resulting alterations in both the inotropic state of the heart, as well as its electrophysiological instability or pro-arrhythmic tendencies [11–16].

The classical studies done on cardiac Purkinje fibres [17] and on mammalian ventricle preparations during ischemia, [18] as well as recent work on the effects of changes in free radical levels on the cardiac Na^+^ current have identified a slowly inactivating component of the Na^+^ current, denoted late I_Na_ or I_Na-L_ (cf [15,19,20]). I_Na-L_ constitutes a potentially quite large source of net Na^+^ influx during cardiac cycle/action potential. This is certainly the case when the ventricular myocardium is challenged (ischemia) or compromised by cardiomyopathy, diabetes, genetic channelopathies etc. [15,20]. Depending upon the pathophysiological setting, the size of this I_Na-L_ can be significant, sometimes reaching approximately 1–3% of the peak Na^+^ current in human ventricle myocytes. This finding, when considered in conjunction with the fact that the I_Na-L_ generates a net Na^+^ influx for almost the entire duration (150–200 msec) of the cardiac action potential plateau raises the possibility that this specific source of net Na^+^ influx may alter [Na^+^]_i_ levels. Indeed, such changes have been demonstrated in isolated Purkinje fibre preparations (c.f. [21]) and in a number of pathophysiological situations using mammalian ventricle preparations [15,20]. In turn, these changes in [Na^+^]_i_ could alter the dynamic balance and/or longer-term homeostatic capacity of one or more of the Na^+^-dependent regulatory mechanisms summarized above. It is also now well recognized that (mainly as a consequence of the very high resistance state of the cardiac myocyte during the entire duration of the action potential plateau) the I_Na-L_ can significantly change the action potential waveform, and may also alter cell-to-cell electrotonic communication [14,22,23].

A growing number of settings in which enhancement of the I_Na-L_ in human ventricle and atria can occur are now significant foci of attention. This interest is based mainly on the modulation of I_Na-L_ by a variety of common pathophysiological stimuli and the resulting alterations in action potential waveform and/or intracellular ion concentrations that can contribute significantly to an enhanced pro-arrhythmic substrate [20]. For example, hypoxia [24,25] short-term ischemic insult [18], free radical challenge [26,27] or related alterations in mitochondrial function that can give rise to free radical species, [28] and thus transiently, but significantly augment, I_Na-L_. In addition, the diabetic myocardium often exhibits an enhanced Na^+^ current as a consequence of changes in tyrosine phosphorylase activity in both Type I and Type II diabetes [29,30]. This increased net inward current produces important changes in the safety factor for action potential repolarization and alters the repolarization reserve [20,22]. The immediate and direct consequences of an increase in I_Na-L_ include a significant lengthening of the action potential duration, sometimes accompanied by (for example in the case of Purkinje tissue) a change in the height of the plateau of the action potential [17,31]. Prominent, but more slowly developing changes in action potential waveform have also been observed [21,32].

At present, the predominant working hypothesis with respect to the sequelae that may lead to the pro-arrhythmic ventricular substrate states that the net Na^+^ influx that corresponds to the enhanced I_Na-L_, can significantly change [Na^+^]_i_, thereby reducing, (perhaps even reversing), Na^+^/Ca^2+^ exchange and resulting in an increase in [Ca^2+^]_i_. In this scheme, augmented [Ca^2+^]_i_ (among other significant effects) promotes phosphorylation of calmodulin, kinase and enhances a number of different ion channel substrates, including Na^+^ and Ca^2+^ channels [20,33,34]. However, we note that previous computational work done to evaluate this hypothesis in settings that are assumed to mimic key elements of human heart failure have also reported only very small changes in [Na^+^]_i_ [14,35,36].

A number of key elements of this working hypothesis have not been evaluated systematically at baseline in human ventricle, i.e., assuming normal electrophysiology and [Ca^2+^] _i_ homeostasis. Accordingly, we have used a current model of the healthy human ventricular action potential [37] and its [Ca^2+^]_i_ homeostasis formalisms to address the following questions:

1. Under control conditions or in the setting of bracketed (2-fold to 5-fold) increases in I_Na-L_, are there significant changes in [Na^+^]_i_ when the heart is paced at physiological rates (1 Hz) or at 2 Hz?
2. Under baseline conditions, and at the highest plausible levels of I_Na-L_, what are the relative contributions to any detectable changes in [Na^+^]_i_ levels, when sources of Na^+^ fluxes, including the Na^+^/K^+^ pump, the Na^+^/Ca^2+^ exchanger, the peak Na^+^ current, and the I_Na-L_ are compared and contrasted?
3. Based on this *in silico* pattern of results: what is the most plausible mechanism by which I_Na-L_ is pro-arrhythmic in human ventricle? How do agents such as ranolazine that function mainly by selective inhibition of the I_Na-L_ result in consistent and important anti-arrhythmic effect therapies?

Our calculations have yielded a pattern of results which demonstrates only quite small changes in [Na^+^]_i_ even following very large (5-fold) and (somewhat implausible) increases in I_Na-L._ These findings suggest that it is *not* solely a progressive increase in [Na^+^]_i_ per se that is the main trigger for pro-arrhythmic changes in the ventricular myocardium. Instead, it is likely that the extraordinary efficacy of I_Na-L_ as a current source (or initiator) for pro-arrhythmia in human ventricles is ‘indirect’ as has been pointed out previously [14,20,22,23].

## Methods

### Human ventricle myocyte action potential model

Simulations of the electrical activity of endocardial human ventricular myocytes were performed using the human ventricular action potential (AP) model developed by O’Hara et al. 2011 [37]. This ‘ORd model’ is based on experimental data taken from 140 healthy human hearts. It includes detailed formulations of 18 ionic currents and provides the possibility for *in silico* studies of endocardial, epicardial, and M cells. The sets of differential equations in this model were implemented in Matlab (Math-works Inc., Natick, MA, USA) and solved numerically using a variable order solver (ode15s).

### Modification of ORd model to simulate [Na^+^]_i_ changes due to I_Na-L_

**Fig 1:**
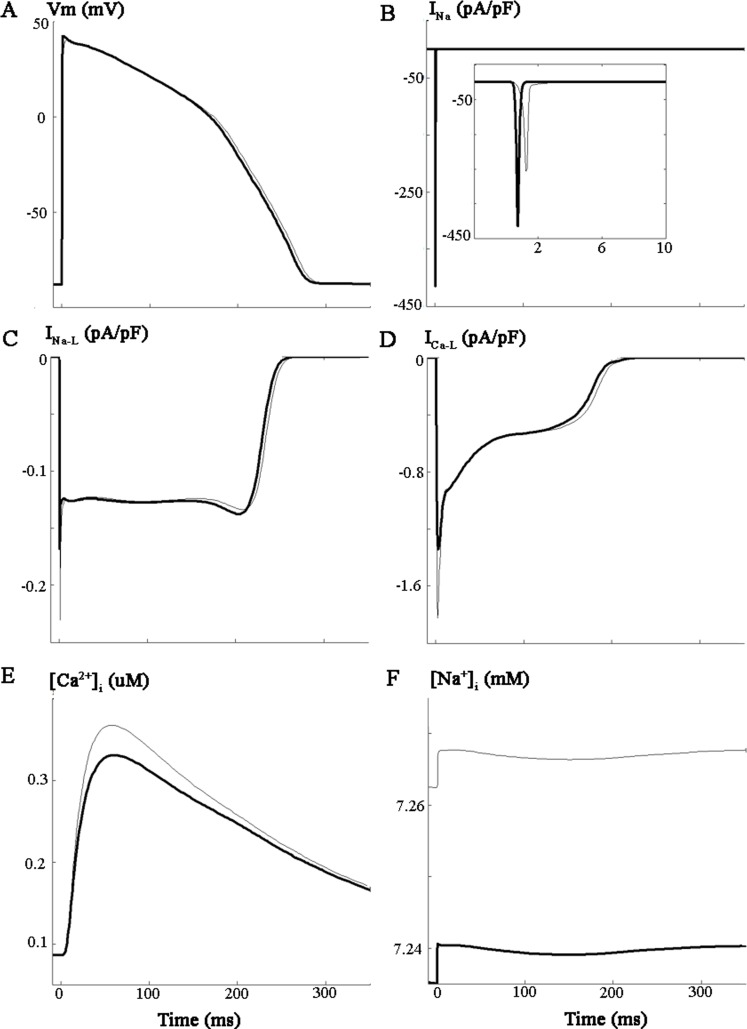
Comparison between the modified and original ORd model of human ventricular AP. Simulations were performed for baseline conditions using the original ORd model (gray traces) and the modified ORd model (black traces) of endocardial human ventricular AP. As described in the methods section, in the modified ORd model, the original formulation for the fast I_Na_ current has beenreplaced with the formulation of ten Tusscher et al. 2004. Simulations were performed at a stimulation frequency of 1Hz, and the last AP (A) after achieving steady state is shown. The following ion currents corresponding to the last pulse are also illustrated: Fast I_Na_ (B), I_Na-L_ (C), I_Ca-L_ (D), as well as [Ca^2+^]_i_ (E) and [Na^+^]_i_ (F).

Our simulations were carried out using a modified version of ORd model in which the equations for fast or peak I_Na_ were replaced with those from ten Tusscher, Noble, Noble and Panfilov model 2004 [38], as suggested by the origninal authors of ORd model to prevent propagation failure (see http://www.ploscompbiol.org/annotation/listThread.action?root=55207). This maneuver was previously tested and validated by ElShrif et al. [39]. In this study, using the TNNP formulation of I_Na_ in ORd model, conduction velocity (CV) yielded values that closely matched experimental data [40] and could also replicate the main characteristics of I_Na,_ restitution properties and spiral wave behavior [39]. To illustrate the behavior of this modified model, Fig 1 shows the comparison of the main ion currents, AP and ion concentrations of the original ORd model and the modified model used in the present study and in [39]. In summary this hybrid ORd model yields a well-characterised experimentally validated simulation modeling environment for the baseline or healthy human ventricular myocyte as judged by the threshold, excitability and the dV/dt of the action potential (cf [39,41]). To ensure that the Na^+^ flux and related change in [Na^+^]_i_ was representative of a range of physiological conditions our simulations were done (i) at baseline, control I_Na-L_ (ii) after increasing I_Na-L_ 2-fold; and (iii) after a 5-fold increase in I_Na-L_.

### Stimulation protocols

Current-clamp simulations were done to evaluate action potential (APs) waveforms and underlying ionic currents, as well as the coincident changes in [Na^+^]_i_ and [Ca^2+^]_i_. Single myocytes were stimulated at either 1 Hz or 2 Hz using supra-threshold depolarizing stimuli (2 ms duration), until steady-state conditions were achieved [42]. Changes in [Na^+^]_i_ at 1 Hz and 2 Hz stimulation rates were evaluated by stimulating myocytes 1000 times at each frequency. [Na^+^]_i_ levels were recorded at: (i) baseline, (ii) after increasing I_Na-L_ either 2-fold or 5-fold, (iii) after inhibition of the Na^+^/K^+^ pump by 50%, (iv) after 2-fold enhancement of the Na^+^/Ca^2+^ exchange current and (v) selected combinations of these conditions.

For the ‘action potential (AP) voltage clamp’ studies, the AP waveform was first recorded from the *in silico* myocyte in current clamp mode (steady-state stimulation at a cycle length of 1 Hz). It was then stored and denoted the ‘short AP’. After I_Na-L_ was increased 5-fold, a ‘long AP’ was recorded and stored. These two AP waveforms were used as voltage clamp commands to evaluate the effects of changes in AP duration and related changes in ionic currents, on [Na^+^]_i_ and [Ca^2+^]_i_. All simulations were done at 1 Hz.

To assess the separate contributions of individual electrogenic currents carried by Na^+^ to the total influx and efflux of Na^+^ and changes in [Na^+^]_i_ during these action potential waveforms, each of these currents (channel, exchange or pump–mediated) was integrated (see Eq 1) during the last action potential in the train.

$$\frac{d{\left[{Na}^{+}\right]}_{i}}{dt}=-(I_{Na}+I_{NaL}+3I_{NCX}{+3I}_{NaK}+I_{Nab})\frac{A_{cap}}{F∙v_{myo}}+J_{diff,Na}\frac{v_{ss}}{v_{myo}}$$ (1)

v_myo_ stands for the bulk myoplasm volume and v_ss_ denotes for the subspace volume (see below).

### Intracellular Volume(s) of Distribution in the ORd Model framework

The ORd model includes four compartments: 1) the bulk myoplasm (myo), 25.84 pL (68% of the cellular volume), 2) the junctional sarcoplasmic reticulum (JSR), 0.182 pL (0.48% of the cellular volume), 3) the network of sarcoplasmic reticulum (NSR), 2.098 pL (5.52% of the cellular volume), and 4) an subsarcolemmal ‘subspace’ (SS) of 0.76 pL (2% of the cellular volume) that represents a microanatomical diffusion-limited space very near the T-tubules. The ORd model also includes distinct Ca^2+^ buffers for each of these compartments (as described in detail in the original paper [37]).

The final part of this study evaluated one mechanism by which I_Na-L_ induced changes in: (i) the action potential waveform and (ii) [Na^+^]_i_ may give rise to early after-depolarizations (EADs). In this set of simulations the base line O’Hara et al, 2011 [37] human ventricular myocyte model was altered. The changes included: i) a reduction in the two main repolarizing K^+^ currents, I_Kr_ (85%) and I_K1_ (30%), and a 3-fold increase in I_Na-L._ Under these conditions when the stimulus rate was reduced to 0.5 Hz, EADs were consistently observed. This combination of changes did not produce EAD-like activity in the ORd under baseline or control AP model (see Discussion).

## Results

**Fig 2:**
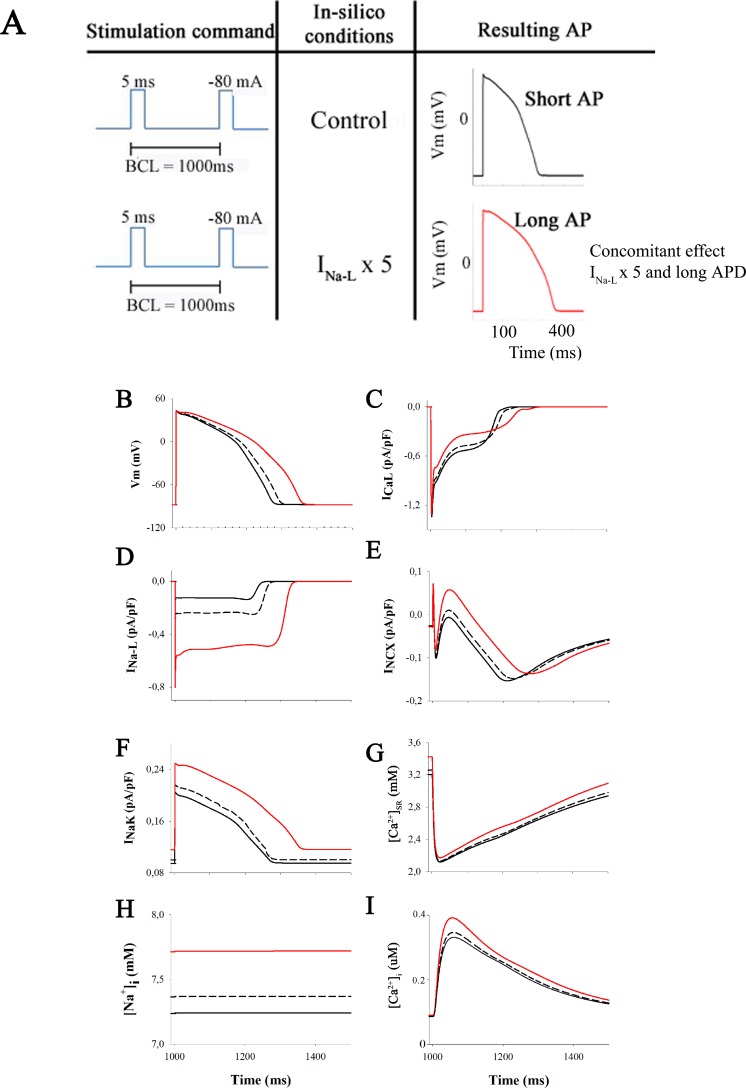
Action potential and ion currents in normal conditions and after I_NaL_ enhancement. Illustration of baseline conditions and steady state effects of increasing late Na^+^ current on the human ventricular action potential and underlying ionic currents. The standard stimulation train lasted 700 s. In this and subsequent Figs the selected conditions are illustrated as follows: control (black lines), 2-fold I_NaL_ increase (discontinuous lines) and then 5-fold I_NaL_ increase (red lines). The stimulation conditions (current clamp) are indicated in panel **A**. The left-hand column shows the 3 action potential waveforms (Panel **B**), corresponding records for I_NaL_ (Panel **D**), electrogenic Na^+^/K^+^ pump current (Panel **F**), and intracellular Na^+^ levels (Panel **H**). The right-hand column shows corresponding data for the L-type Ca^2+^ current (Panel **C**), the Na^+^/Ca^2+^ exchange current (Panel **E**), a parameter depicting the amount of Ca^2+^ release from the sarcoplasmic reticulum (SR) (Panel **G**) and the resulting intracellular Ca^2+^ levels (Panel **I**).

The starting point (or baseline conditions) for this set of simulations is illustrated in Fig 2 (black traces). Panel A shows the stimulation command, the simulation conditions and the resulting APs. Panel B to I illustrate the steady-state action potential waveform (B), the corresponding L-type Ca^2+^ current (C), late Na^+^ current, I_Na-L_ (D), Na^+^/Ca^2+^ exchanger current I_NCX_ (E), Na^+^/K^+^ pump current I_Na-K_ (F), total sarcoplasmic reticulum and intracellular Ca^2+^ levels [Ca2^+^]_SR_, [Ca^2+^]_i_, (G) and (I), and intracellular Na^+^ levels, [Na^+^]_i_ approx, 7.25 mM in our model of the healthy human ventricle myocyte (H).

To begin to evaluate whether Na^+^ influx through I_Na-L_ can alter [Na^+^]_i_, two sets of simulations were done. First, I_Na-L_ was increased 2-fold to approximate the changes in I_Na-L_ utilized in a number of different experimental settings that have evaluated repolarization variability caused by changes in I_Na-L_ [3,23]. These results are shown as hatched black lines, superimposed on our baseline simulations. Note that in response under steady-state conditions: (i) the action potential lengthens substantially (A), (ii) [Na^+^]_i_ increases by only approx. 0.1 mM (D); and (iii) there are small changes in the Na^+^/K^+^ pump current (C), as well as the L-type Ca^2+^ current I_Ca-L_ (E) and the Na^+^/Ca^2+^ exchanger current, I_NCX_ (F).

The second set of simulations was done to evaluate the effects of increasing I_Na-L_ 5-fold. This is an extreme maneuver; well beyond the fold changes reported in even pathophysiological settings. However, it is somewhat similar to some of the experimental results obtained after pre-treatment of ventricular myocytes with a sea anenome toxin, ATX II that selectively augments I_Na-L_ (cf [20]). As illustrated by the red traces, in each panel of Fig 2 the two most notable and functionally significant changes are: (i) an increase in [Na^+^]_i_ by approx. 0.5 mM to approx 7.7 mM (D) and corresponding increases in (ii) the Na^+^ /K^+^ pump current (C) and (iii) I_NCX_ (F).

**Fig 3:**
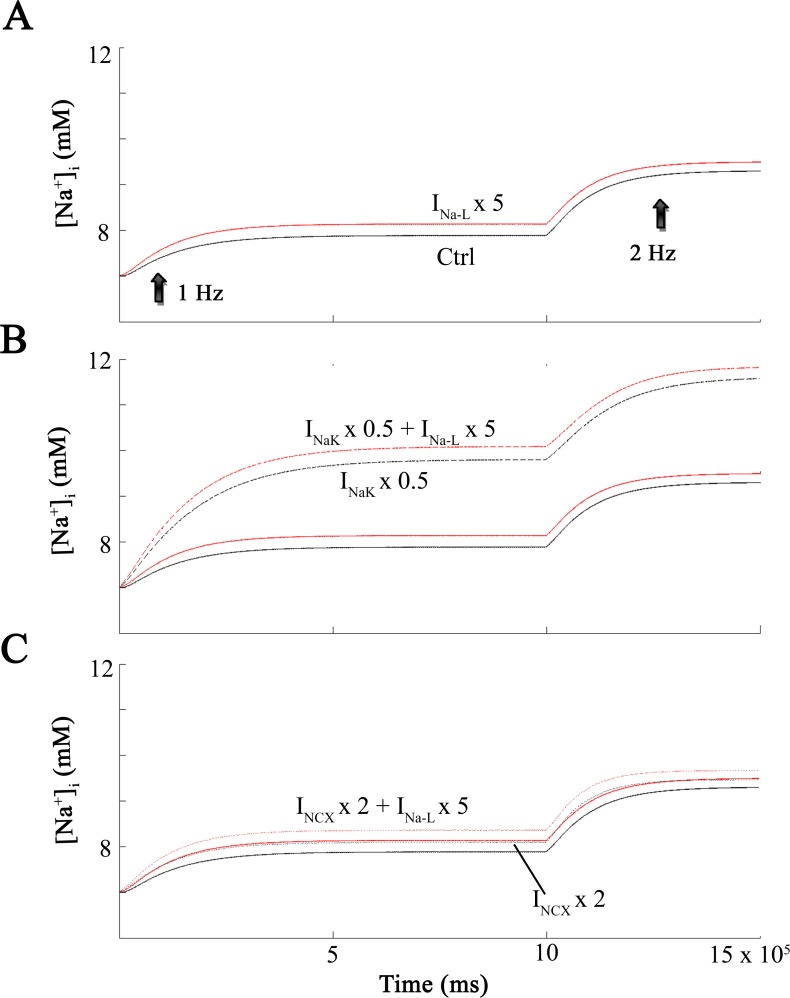
[Na^+^]_i_ levels at increasing frequencies. Demonstration of intracellular Na^+^ levels ([Na^+^]_i_ during maneuvers that form the basis of this paper. Each *in silico* test involved the application of 1000 stimuli applied at a rate of 1 Hz (left) and then 2 Hz (right). This maneuver was done under baseline conditions (black) and also after increasing I_NaL_ 5-fold (red). Effects on [Na^+^]_i_ are shown in Panel **A**. The remaining data sets correspond to 50% inhibition of I_NaK_ (discontinuous black curve) and 50% inhibition of I_NaK_ and 5-fold increase of I_NaL_ (discontinuous red curve) in Panel **B**. In Panel **C** the black dotted curve corresponds to a 2-fold increase in I_NCX_, and the dotted red curve corresponds to a 2-fold increase in I_NCX_ and a 5-fold increase in I_NaL_.

With this pattern of results as a guideline, we next explored the time course and the extent of changes in [Na^+^]_i_ after a 2-fold increase in heart rate (cf [43]) under many of the same conditions that are illustrated in Fig 2. The two superimposed traces in Fig 3A show [Na^+^]_i_ at baseline (black) and also in response to stimulus trains consisting of 1,000 action potentials applied first at 1 Hz and then at 2 Hz. The red trace in this Panel illustrates results produced by an analogous set of simulations, except that I_Na-L_ was increased 5-fold.

The superimposed [Na^+^]_i_ data in Panel 3B shows the effects of reducing Na^+^/K^+^ pump activity by 50% under the conditions where I_Na-L_ was also increased 5-fold. Again, results were obtained at two stimulation rate: at 1 Hz and 2 Hz. Note that at 1 Hz [Na^+^]_i_ reaches a maximum of approx 10 mM (red trace). The corresponding [Na^+^]_i_ value for the 2 Hz stimulation is 12 mM (dashed red line). When [Na^+^]_i_ increases to either approx.10 or 12 mM the Na^+^/K^+^ pump is strongly stimulated, as expected [4,44] and this limits the extent of the increase in [Na^+^]_i_. To visualize the effects of the 2 Hz stimulus train in the absence of the enhanced activation of the electrogenic Na^+^/K^+^ pump activity, simulations were also done with the Na^+^/K^+^ pump ‘blocked’ by 50%. As shown by the superimposed traces in Fig 3B the resulting increases in [Na^+^]_i_ were somewhat larger when Na^+^/K^+^ pump activity was reduced by 50%. The traces in Panel C show the changes in [Na^+^]_i_ when the 1 Hz and 2 Hz stimuli are applied at baseline and then in the presence of a (i) 5-fold increase in I_Na-L_, and also (ii) with the Na^+^/Ca^2+^ exchange activity increased 2-fold (see Discussion).

**Fig 4:**
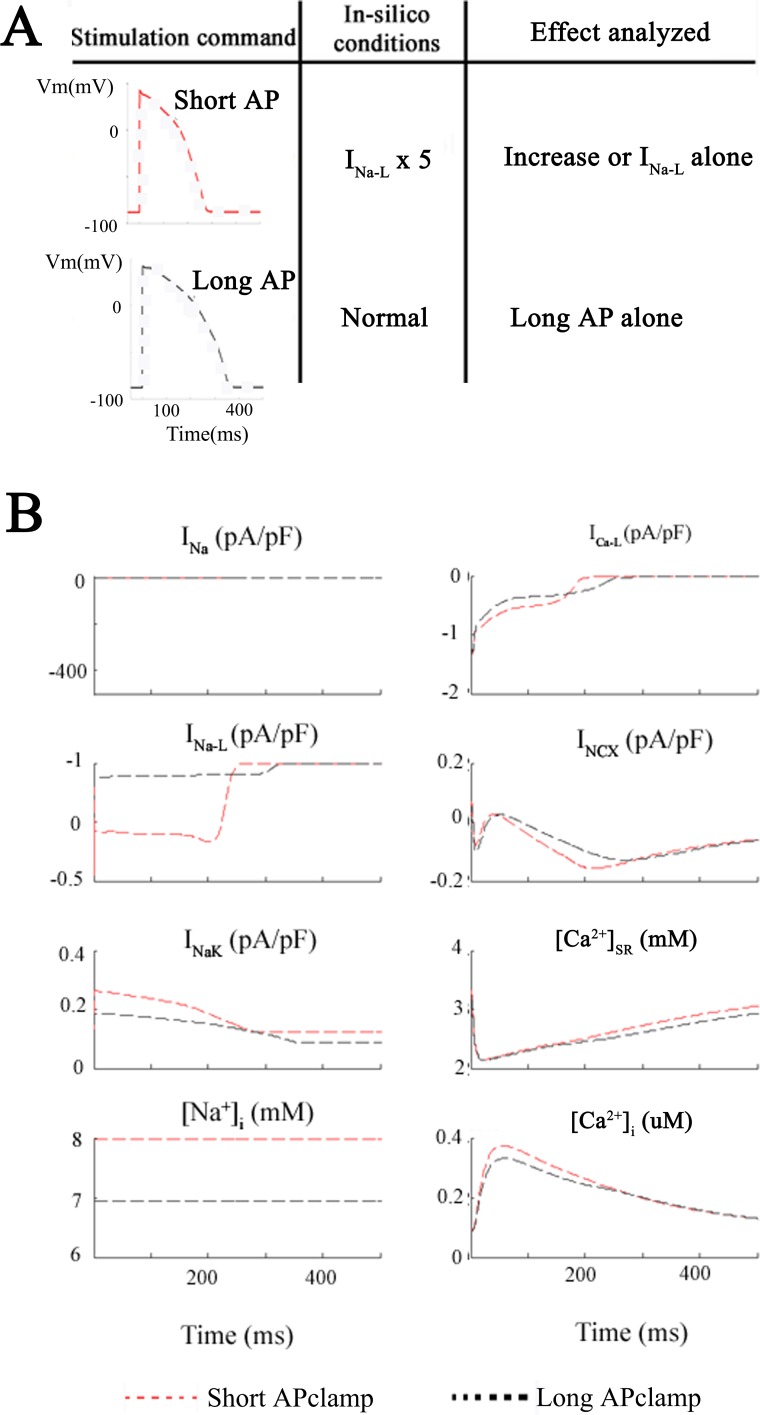
Action potential clamp simulations. Analysis of the effects of enhancing I_NaL_ 5-fold under conditions where the action waveform is held or ‘clamped’ at its control or baseline waveform. Panel **A** shows this protocol. First simulations were run with 5x-I_Na-L_ but with a fixed or “clamped” normal AP waveform (discontinuous red lines). Thereafter, the long AP waveform due to 5x-I_Na-L_ was applied as the voltage clamp command signal (discontinuous black lines) and I_NaL_ remained at its baseline value. The resulting changes in 8 selected parameters are plotted in Panel **B**. Based on this pattern of results, it is clear that the small increase in [Na^+^]_i_ from 7 to 8 mM is due to the Na^+^ influx through I_NaL_ at baseline i.e. that the change in AP waveform is not a significant factor.

It is well known that significant increases in I_Na-L_ can change the action potential waveform, mainly by lengthening its duration (APD), and increasing its height during the plateau phase (cf. [17]). However, in the healthy human ventricular myocardium there has been no previous attempt to separate these changes in order to address the questions: Is it: (i) the Na^+^ flux due to I_Na-L_ per se, (ii) the concomitant change in action potential waveform, or (iii) both, that enhance the likelihood of pro-arrhythmic activity in human ventricle? The superimposed sets of results shown in Figs 1 and 4 provide much of the data that is needed to address these points. As described above, two of the sets of superimposed traces in Fig 2 were obtained: (i) under baseline conditions (1 Hz, black traces) and (ii) 1 Hz, with I_Na-L_ enhanced 5-fold (red traces).

The next set of simulations employed the action potential waveform as the activating stimulus (Fig 4). This was done to simulate action potential clamp methodology. By selecting representative ‘short’ or ‘long’ AP waveforms (see Methods) as the command signal for the *in silico* voltage clamp, one can separate the effects of (i) action potential lengthening and (ii) an enhanced I_Na-L_ on corresponding changes in [Na^+^]_i_. In Fig 4 the baseline action potential (denoted ‘short AP’ and illustrated as the discontinuous red traces) was the digitized waveform shown in black in Panel B of Fig 2. The long action potential (discontinuous black trace) was the digitized AP corresponding to the red trace in Fig 2B. Importantly, these simulations show that the [Na^+^]_i_ increase is only slightly larger (approx. 1 mM) when I_Na-L_ is enhanced 5-fold and the short AP waveform is used (discontinuous red trace: short AP + 5×I_NaL_), than when the long AP waveform is applied, but I_Na-L_ is maintained at its baseline value (discontinuous black trace: long AP + 1×I_NaL_). In summary, even very substantial action potential lengthening does *not* appear to be a primary factor in markedly changing either [Na^+^]_i_ or [Ca^2+^]_i._ Rather, an enhancement of I_Na-L_ *per se* plays a more important role in [Na^+^]_i_ changes. [23](cf Discussion).

### Relative contributions of Channel, Pump and Exchanger Mechanisms to [Na^+^]_i_ Homeostasis in Human Ventricle

**Fig 5:**
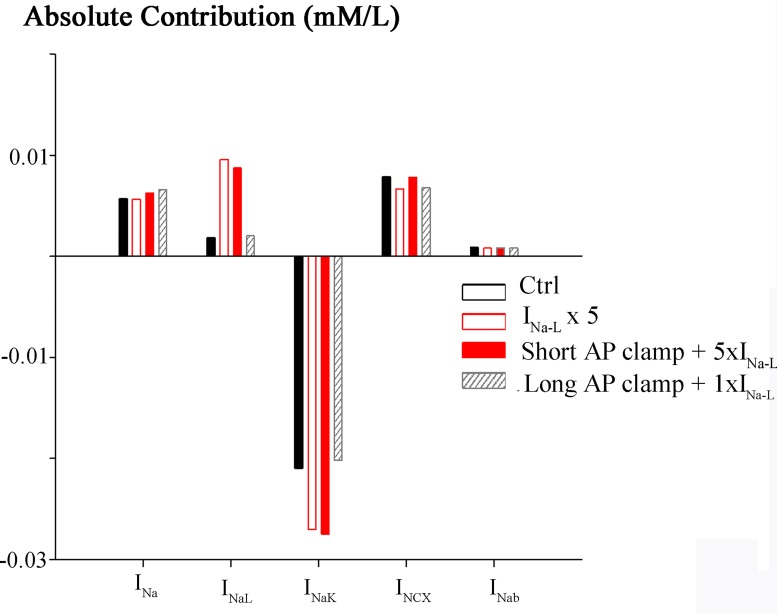
Absolute Na^+^ fluxes. Contribution of the selected electrogenic currents carried by Na^+^ to the total influx and efflux of Na^+^ during the human ventricular action potential. To obtain these results, each channel, exchanger or pump-mediated Na^+^ flux was integrated during the last action potential. All simulations were conducted at 1 Hz. The black bars reflect values for steady-state control conditions (current clamp). The white bars outline in red correspond to a 5-fold increase in I_NaL_ (current clamp) i.e. where the AP is long. In this case, both i.e. an enhanced I_NaL_ as well as the long AP are considered. The solid red bars correspond to short AP clamp, where I_NaL_ was enhanced 5-fold (short AP + 5×I_NaL_). In this case therefore only the effect of on [Na^+^]_i_ an enhanced I_NaL_ is analysed. Hatched bars correspond to long AP clamp, with normal I_NaL_, so that the effect of a long AP alone is illustrated (long AP + 1×I_NaL_).

**Fig 6:**
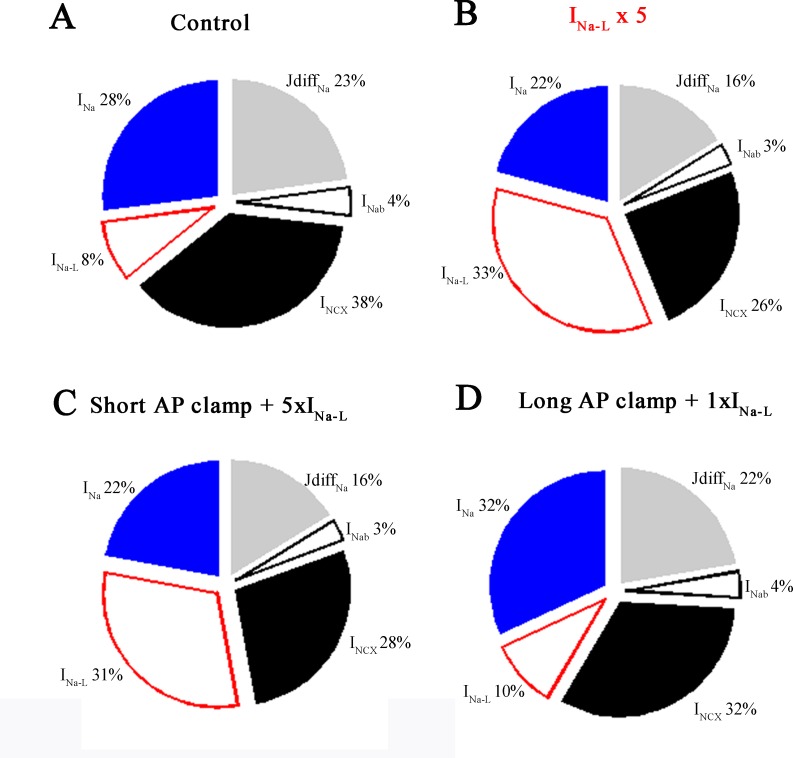
Relative Na^+^ fluxes. The relative contributions of selected Na^+^ flux pathways to [Na^+^]_i_ homeostasis. These relative flux values were calculated during the last AP (number 700) for each of the four conditions described in Figs 2 and 4. Simulations were conducted at 1 Hz: (**A**) steady-state control conditions (current clamp), (**B**) a 5-fold increase in I_Na-L_ (current clamp), (**C**) short AP clamp and a 5-fold increase in I_Na-L_ (short AP + 5×I_NaL_), and (**D**) long AP clamp with normal I_Na-L_ (long AP + 1×I_NaL_).

These computations can also reveal the relative contribution of each of the Na^+^ dependent ion transport or processes that are operative in this model of human ventricular myocyte Na^+^ homeostasis. [1,5,6,45,46] A synthesis of this information is presented in Figs 5 and 6. The integrated Na^+^ flux per action potential is illustrated under each of the selected conditions of this study (Figs 2–4). The histograms shown in black in Fig 5 were obtained under control (or baseline) conditions, that is, at steady state following a train of 1 Hz stimuli. Each separate electrogenic Na^+^ flux was integrated during the last action potential of the AP train. Note that during the action potential at baseline the net Na^+^ influx due to the peak Na^+^ current is considerably larger than that due to I_Na-L_. The corresponding histograms illustrating Na^+^ fluxes mediated by the Na^+^/K^+^ pump and Na^+^/Ca^2+^ exchange also provide useful insights. For example, the Na^+^/K^+^ pump generates a substantial Na^+^ mediated outward current, which corresponds to: (i) the approximate Na^+^ influxes due to the peak and late components of the Na influx due to I_Na,_ (ii) as well as baseline influx of the Na^+^/Ca^2+^ exchanger. The Na^+^ influx due to the assumed (very small) background Na^+^ current is also illustrated. An analogous data set, obtained under the same conditions except that I_Na-L_ was increased 5-fold is shown in the histograms that are outlined in red. Aside from documenting that the I_Na-L_ has been increased 5-fold, there are no significant new insights from this maneuver.

The third data set was obtained using the baseline or ‘short’ action potential clamp, together with the 5-fold increase in I_Na-L_ (red histograms) As expected, the increase in [Na^+^]_i_ resulting from this enhanced Na^+^ influx stimulates the Na^+^/K^+^ pump. Interestingly, it has no other significant effect on the overall net fluxes generated by these Na^+^ dependent transport mechanisms. Importantly, the Na^+^/Ca^2+^ exchanger continues to operate in the forward mode. The final data set in this Figure (histograms that are cross hatched) shows the effects of lengthening the action potential in the presence of a baseline level of Na^+^ current. This maneuver (by itself) produces no significant changes in peak Na^+^ current, I_Na-L_, Na^+^/K^+^ pump current, or Na^+^/Ca^2+^ exchange current.

An additional way of considering the results from these simulations and evaluating their implications is to summarize the relative changes in Na^+^ homeostasis in the form of pie charts that depict the relative fluxes mediated by each of these Na^+^ dependent transport mechanisms. The data shown in Fig 6A was obtained by integrating the Na^+^ fluxes during the last action potential of a 700 action potential train elicited at 1 Hz. Note that the Na^+^ flux due to the peak Na^+^ current is approximately 3.5 times larger (28% vs. 8%) than that due to I_Na-L_; and also that the Na^+^ flux generated by the I_NCX_ is the largest of these three electrogenic Na^+^ transport mechanisms. The analogous data set in Fig 6B shows these proportional fluxes and relative changes when I_Na-L_ was increased 5-fold. As expected, the Na^+^ flux mediated by I_NaL_ increases substantially (approximately 4-fold). The Na^+^ influx due to peak Na^+^ current changes very little. The significant decrease in Na^+^ flux mediated by the I_NCX_ (38% vs. 26%) is due to the very long plateau of the action potential under these conditions. The intrinsic, non-linear voltage dependence of I_NCX_ mediated ion transport results in this current being much smaller at membrane potential corresponding to the action potential plateau than at diastolic membrane potential (see Discussion).

Comparison of the data in Fig 6D and 6A provides further basis for determining whether in fact, the lengthening of the action potential by itself is a significant factor in [Na^+^]_i_ accumulation in healthy human ventricular myocytes. Apparently, this is not the case, based on the similarity of these relative fluxes under these two very different sets of conditions. In summary, under these conditions, as was the case at baseline, the major pathways for Na^+^ flux are the peak Na^+^ current and the Na^+^ flux mediated through the I_NCX_. The Na^+^ flux due to the I_Na_-_L_ current increases [Na^+^]_i_ only approximately 2%. This may seem unlikely, (even counter intuitive); however, it is important to recognize that the long AP waveform corresponds to an action potential plateau at approximately +30 mV, (very close to the electrochemical equilibrium potential for Na^+^). Given this, lengthening of the ventricular action potential may not result in any significant increased Na^+^ influx (see Discussion).

## Discussion

### Summary

The most significant insight gained from this set of computations is that within the framework of the O’Hara et al, 2011 [37] model of electrophysiological activity at baseline (in control conditions) in the healthy human ventricle, even very strong activation of the I_Na-L_ in single myocytes results in only an approx. 1 mM increase in intracellular Na^+^, [Na^+^]_i_ at a physiological (1 Hz) heart rate. In fact, under these conditions (see Fig 2), with I_Na-L_ adjusted within the physiological range (from baseline to a two-fold increase, there is apparently no detectable (≤0.2 mM) increase in [Na^+^]_i_. We are not the first Group to have reached this conclusion based on mathematical modeling of Na^+^ fluxes in mammalian ventricular myocytes. A number of recent papers [3,14,23,36] suggest this although in most cases the context is the failing ventricle, and thus a very different electrophysiological substrate [35]. Another important finding, of this work, not tested to date, is that the effect of I_Na-L_ on [Na^+^]_i_ elevation is direct and not derived from APD prolongation.

Our conclusion/illustration does, however depend on the assumption that the Na^+^ influx due to I_Na-L_ is distributed quasi-instantaneously into approx 50% of the total or right-cylindrical volume of the myocyte (see Methods). Evidence in favor of a small intracellular ‘fuzzy space’ in which Na^+^ could accumulate, would be expected to enhance the extent to which [Na^+^]_i_ can rise e.g. after a train of action potentials at a high heart rate [47–50]. However, the increase in [Na^+^]_i_ in this ‘fuzzy space’ would be transient, since Na^+^ is highly mobile/diffusible in myoplasm [44]. Nonetheless, transient increases of [Na^+^]_i_ in the 2–5 mM range could have some very significant effects, even if the reduction in the electrochemical gradient for [Na^+^]_i_ was not large enough to reverse Na^+^/Ca^2+^ exchange flux [48,51]. For example, if [Na^+^]_i_ rose by e.g. 5 mM the Na^+^/K^+^ pump activity (turnover rate) would increase and this could have secondary metabolic consequences, perhaps even leading to altered ATP/ADP ratios [52]. Activation of the eletrogenic Na^+^/K^+^ pump would also hyperpolarize the myocyte resting membrane potential [4,45,53,54] by e.g. 3–5 mV. This may alter excitability by partially removing I_Na_ inactivation. In pathophysiological settings, e.g. in the cardiomyopathic [35,55] or in failing hearts [45,56–58] where I_Na-L_ is increased and the input resistance of the myocytes, (even at diastolic membrane potentials), is also increased; enhanced activity of the electrogenic Na^+^/K^+^ pump would be expected to produce a somewhat larger hyperpolarization of the diastolic membrane potential (assuming that the expression density and Na^+^/K^+^ pump isoform remain unchanged) [59–61]. In the setting of free radical challenge in the failing heart, I_Na-L_ is increased substantially [27] while Na^+^/K^+^ pump turnover is reduced [62]. This combination of conditions would pre-dispose the human ventricular myocardium to exhibit [Na^+^]_i_ ‘accumulation’ or overload [62–64]. However, as mentioned, previous assessments of [Na^+^]_i_ in ‘failing human ventricular myocytes have also not revealed any significant I_Na-L_ dependent increase in [Na^+^]_i_ [3,14,20,35,64].

Increased [Na^+^]_i_ could also reduce Na^+^/H^+^ and/or Na+/HCO_3_^-^ exchanger activity. The resulting compromise of intracellular pH regulation [7] is known to have significant effects on both channel-mediated currents and intracellular communication/conduction [8]. The Backx Group [65] have shown that increases in [Na^+^]_i_ from baseline approx. 5–7 mM to approx. 10–12 mM can significantly alter myocyte contractility. This occurs mainly by enhancing Ca^2+^-induced Ca^2+^ release. Previously, Levi et al [66] had reported somewhat similar results.

### I_Na-L_ as a target for anti-arrhythmic agents

To fully understand the impressive anti-arrhtyhmic action of selective I_Na-L_ blockers, it is helpful to dissect the negative effects of I_Na-L_ enhancement on Na^+^, Ca^2+^ handling, and AP waveform, in the healthy and in the pathological myocardium. Many studies focusing the role of I_Na-L_ enhancement in arrhytmogenesis have been done under pathological conditions, such as heart failure or hypertrophic myocardiopathy [67]. Under such circumstances, Na+ accumulation, abnormal Ca^2+^ handling, APD prolongation, EADs and DADs generation cannot be only attributed to I_Na-L_ enhancement. Remodeling of other ion currents and metabolic changes are also responsible for these changes.

**Fig 7:**
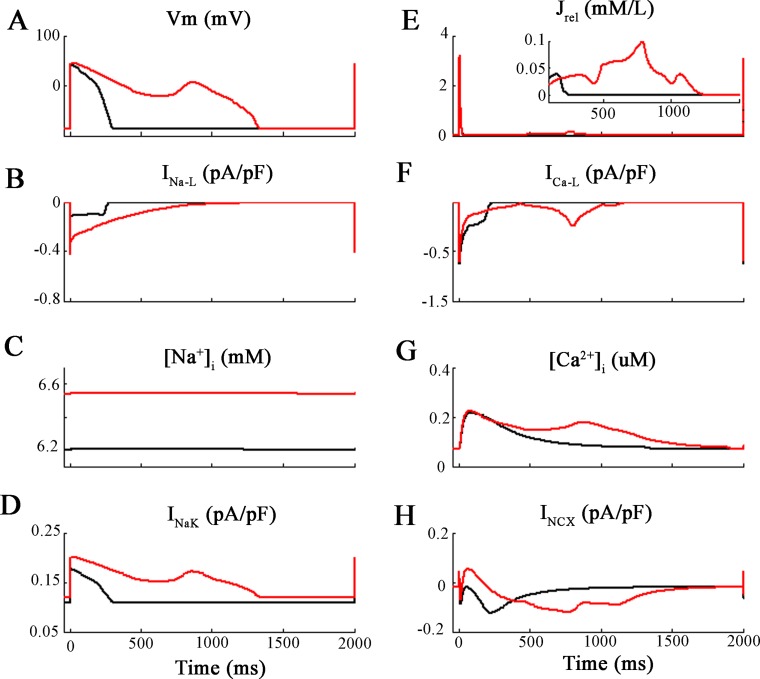
Early after-depolarizations (EAD’s) using the modified ORd model of (endocardial) human ventricular AP. Simulations were performed for baseline conditions (black traces), and also after a three-fold increase of the late sodium current (I_Na-L_) (i) This change coupled with a set of other parameter changes yielded consistent EAD generation at a slow (0.2 Hz) stimulus rate. The changes were: 80% block of the rapid delayed rectifier current (I_Kr_), plus (ii) 30% block of the background inwardly rectifying K^+^ current (I_K1_) (red trace). Panels A illustrates the AP waveform and EAD Panels B and F show the late sodium current, I_Na-L_ and Ca^2+^ current (I_Ca-L_) respectively. Panels C and G illustrate [Na^+^]_i_ and [Ca^2+^]_i_ levels. Panels D and H depict Na^+^/K^+^ pump and Na^+^/Ca^2+^ exchange currents.

In this work, the effect of I_Na-L_ enhancement was analyzed in healthy human ventricular myocytes. The first and well characterized effect of I_Na-L_ enhancement is APD prolongation, in agreement with experimental works where specific mutations lead to I_Na-L_ enhancement [68]. Here resides the classical explanation for the impressive anti-arrhythmic action of agents such as ranolazine that quite selectively reduces I_Na-L_ in mammalian ventricle, including humans [15,34]. APD prolongation is proarrhythmic because it can result in reactivation of the I_Ca-L_ current [69,70]. Based on our simulations, this mechanism appears to be plausible. Although a number of significant changes in K^+^ current(s) need to be implemented simultaneously. These included: (i) I_Na-L_ increased 3-fold, (ii) stimulation rate reduced to 0.5 Hz and (iii) repolarization reserve markedly decreased by reducing HERG by 85%; and also reducing the background K^+^ current I_K1_, by 30%. The resulting EAD activity is illustrated in Fig 7. We note that the L-type Ca^2+^ current in ventricular myocardium also exhibits a slowly inactivating or ‘late’ component; and this has been shown to contribute to formation of EADs [71]. This combination of maneuvers is quite extreme and certainly non-physiological. However, it is not unprecedented within an electrophysiological substrate thought to be representative of failing ventricular myocardium (cf. [67,72–75]). It is therefore not surprising that discussion, controversy, and disagreement persist regarding the physiological and pathophysiological consequence of I_Na-L_ [16,76–79].

The second effect of I_Na-L_ enhancement observed in our simulations is the slight [Na^+^]_i_ increase. In the healthy myocardium, I_Na-L_ apparently does not significantly alter [Na^+^]_i_, even when it is increased 5-fold and heart rate is doubled for 700 beats at 1 Hz. This raises the question: What is a plausible mechanism for the anti-arrhythmic action of ranolazine? I_Na-L_ enhancement could also be considered proarrhythmic, because of the effects on [Na^+^]_i_ elevation. Indeed, our AP clamp simulations showed that APD prolongation per se did not elevate [Na^+^]_i_, whereas I_Na-L_ enhancement with a clamped short AP (short AP + 5×I_NaL_) did elevate [Na^+^]_i_. Thus, the slight increase in [Na^+^]_i_ under conditions of enhanced I_Na-L_, is a direct effect of this current through the increase of Na^+^ flux, rather than an indirect effect related to APD prolongation. A more significant [Na^+^]_i_ elevation, under specific conditions, such as hypertrophic cardiomyopathy [35] might be proarrhythmic through Ca^2+^ overload and ranolazine can reverse these effects.

Finally, the third arrhythmogenic effect of I_Na-L_ enhancement is an abnormal Ca^2+^ handling, which is very much related to the two effects described above: Na^+^ accumulation and APD prolongation. Our AP clamp simulations showed that in the healthy myocardium, I_Na-L_ enhancement slightly increased peak [Ca^2+^]_i_. This has also been observed in experimental studies on mice myocytes [68] in a more pronounced manner. Another important finding from our AP clamp simulations is that APD prolongation per se did not significantly alter [Ca^2+^]_i_ transient. This observation differs from Bouchard et al. [80] results, who performed AP clamp experiments in rat myocytes and observed that a rapid change from a “short” to a “long” action potential command waveform resulted in an immediate decrease in peak I_Ca-L_ and a marked slowing of its decline. Prolongation of the action potential also resulted in slowly developing increases in the magnitude of Ca^2+^ transients. They suggested that a change in Ca^2+^ content of the sarcoplasmic reticulum was responsible for this effect, and that changes in the rate of rise of the [Ca^2+^]_i_ transient were closely correlated with changes in the magnitude and the time course of I_CaL_. Thus, Ca^2+^ release from the sarcoplasmic reticulum can be modulated by the action potential waveform as a result of changes in I_Ca-L_. In our case, the longer AP in Fig 1 caused a slight decrease of I_CaL_ although the current is active for a longer time. Thus, Ca^2+^ influx should not be significantly. Differently, changes in AP upstroke driven by an increase of I_Na_ peak in ORd model lead to a decrease of I_CaL_ peak and a subsequent decrease of [Ca^2+^]_i_ peak.

In summary, changes in I_Na-L_ do affect Ca^2+^ handling, as showed our simulations and several experimental studies [68], and changes in AP waveform per se might affect Ca^2+^ handling depending on how ca currents are affected by the AP change. It would be very interesting to perform AP clamp experiments on human myocytes similar to Bouchard et al. [80] experiments to elucidate the effects of AP waveform changes on Ca^2+^ handling. With respect to the effects of I_Na-L_ enhancement on Ca^2+^ handling under pathological conditions, many authors have demonstrated the derived arrhythmogenic events and the power of I_Na-L_ blockers to reverse these situations [14,67]. Again, in these cases many factors rather than I_Na-L_ alone are to be considered.

### Limitations of this study

Our analysis and conclusions must be considered in the context of the strengths and limitations of the chosen mathematical modeling platform, the O’Hara et al. [37] model of the baseline or human ventricular action potential and its intrinsic intracellular Ca^2+^ homeostasis mechanism(s). As has been pointed out previously, our choice of the model is well-founded. Thus i) this model was constructed using data from a large number of human ventricle myocyte preparations (n = 140) that were derived from healthy donors, and ii) it has been relatively well characterized through modifications/further testing by the Rudy Group and extensive use by others, including being adopted for the modeling platform of the FDA Safety Pharmacology initiative. Nonetheless:

1. The original equations for I_Na_ in this model give rise to a Na^+^ current that promotes only a marginal safety factor for depolarization and a relatively low conduction velocity. As noted we have therefore exchanged the original O’Hara et al [37] formulation for I_Na_ with the analogous equations from ten Tusscher et al [81]. The size of I_Na-L_ in the healthy human ventricular myocyte was approximated under control conditions and then both 2x and 5x increases were evaluated to ensure that the resultant Na^+^ influxes and related changes in [Na^+^]_i_ bracketed all plausible physiological conditions. The changes in I_Na_ formulation, also adopted by other authors [39,82], slightly alters the AP waveform, I_CaL_, and Ca^2+^ handling (see Fig 1). A higher I_Na_ leads to a faster depolarization phase and a more depolarized overshoot of the AP. Because of I_CaL_ voltage dependence, I_CaL_ peak decreases and plays a smaller role in the depolarization phase. Some authors have shown experimentally and theoretically that changes in fast I_Na_ directly affect I_CaL_ behavior. For instance Shaw and Rudy [83] used their AP model for guinea-pig ventricular myocyte to show that under conditions of hyperkalemia, when I_Na_ availability is reduced, the AP upstroke is “taken over” by I_CaL_, which substantially increases its peak. Also Verheijck et al. [84] in evaluating the contribution of I_CaL_ current to depolarization of sinoatrial nodal myocytes of rabbits, stated that inhibition or stimulation of a single pacemaker current inevitably influences the other pacemaker currents. It is to be noted that any change in AP waveform affects I_CaL_. Saegusa et al. [85] performed experiments on rabbit ventricular myocytes and showed that intracellular acidosis strongly decreased I_to_, increasing AP plateau and decreasing peak I_CaL_. Their AP voltage clamp approach allowed them to determine that intracellular pH-induced changes in AP waveform (plateau elevation) per se, reduced I_CaL_, without the complicating effects of intracellular acidosis. Similarly, APD prolongation increased the net Ca^2+^ influx, affecting Ca^2+^ handling.
2. Although this O’Hara et al [37] model provides the possibility of assessing known or suspected pro or anti-arrhythmic effects that may arise from predominant or selective effects on epi-versus endocardial ventricular ‘myocytes’ our simulation made are of only the endocardial myocytes model. We acknowledge that there is evidence that in the healthy ventricular myocardium I_Na_ is distributed in a heterogeneous fashion. I_Na_ is larger in the endocardium [86,87] and it was for this reason that our simulations were done using the endocardial myocyte model. With respect to pro-arrhythmic sequelae, it would be of interest and importance to explore, in detail, transmural dispersion as a pro-arrhythmic factor.
3. More accurate computations of the Na^+^ fluxes due to both the peak and the I_Na-L_ in human ventricle should be made after re-formulation of the Na^+^ equations to account for the fact that this channel (and virtually all others) do **not** function as a perfectly Na^+^ selective ion transport mechanism under either physiological or pathophysiological conditions [88]. Rather, at positive membrane potential including those that are representative of the plateau of the ventricle action potential (in either the nonconducted or membrane mode or during AP propagation) there is a significant K^+^ efflux through the Na^+^ channel pore complex. This changes the apparent reverse potential for I_Na_ from e.g. +65mV to +50mV and alters action potential overshoot and plateau height accordingly. This quite small change could alter the I_Na-L_ waveform; and the likelihood that I_CaL_ is reactivated. Indeed, an important and well characterized arrhythmogenic effect of changes in AP waveform is the reactivation of I_CaL_, precursor for EAD formation [89]. Karagueuzian et al. [90] pointed out in their review article the role of the I_CaL_ window current, which can reactivate between -40 mV and 0 mV causing EAD formation under circumstances of oxidative stress, when repolarization reserve is reduced and AP waveform is affected. Moreover, Ca^2+^ release from the SR is known to be strongly dependent on the AP waveform in general, and the repolarization profile, in particular. [80,91]
4. Any insights into plausible mechanisms based I_Na-L_ being an important site for anti-arrhythmic therapies are also ‘model-dependent’. Thus the intracellular Ca^2+^ transient in the O’Hara model exhibits only a rather limited dynamic range; that is, resting [Ca^2+^]_i_ as approximately 0.1 μM, and at peak systole [Ca^2+^]_i_ is approximately 0.3 μM. A larger, and in fact more realistic, physiological change in [Ca^2+^]_i_ is likely to produce a secondary release of Ca^2+^ from the SR and this is known to contributed to both EAD and DAD formation as a result of this Ca^2+^-induced augmentation of the electrogenic Na^+^/Ca^2+^ exchanger [70,92,93]. One example of an interesting and important refinement in the context that should be considered is the following: in a detailed study of the effects of changes in [Na^+^]_i_ an SR-mediated Ca^2+^ release Ramirez et al [65] have reported that increase in [Na^+^] in the 5–15 mM range augments the Ca^2+^- induced Ca^2+^ release process, perhaps due to ‘reverse-mode’ Na^+^/Ca^2+^ exchange (although these experiments were done using adult rat ventricular myocytes).
5. As mentioned, our assessments of transient or steady-state changes in [Na^+^]_i_ and those published previously (cf [3,14,36]) are all based on the assumption that the Na^+^ influxes due to both peak I_Na_ and I_Na-L_ are distributed instantaneously and uniformly into an intracellular compartment that corresponds to approximately 50% of the entire right-cylindrical volume of the human ventricular myocyte (see Methods). That is, the only intracellular volumes that are not freely accessible are those occupied by intracellular organelles (nucleus, mitochondria, SR, contractile proteins). In the steady-state this reasoning is sound; however, under specific conditions (e.g. after each action potential) [Na^+^]_i_ could be distributed temporarily in a ‘putative fuzzy space’ (c.f. [47]). In addition, [Na^+^] homeostasis in the myoplasm may differ substantially from that in the mitochondria and substances released by the mitochondria (e.g. H_2_0_2_ may alter I_Na-L._ [46,94]. Furthermore, the presence of Na channels within the t-tubules has been well documented both structurally as well as functionally by multiple investigators [95]. Placing Na channels in t-tubules in the model might allow the local rise in Na^+^ and might affect close L-type Ca^2+^ channels and Ca^2+^ dynamic. However, ORd model does not consider this distribution of Na^+^ channels, which would require cautious modifications in the model. Indeed, a human detailed AP model including more accurate models of t-tubules, as in the model of rat myocytes by Pasek et al. [96] would be very valuable to analyze Na^+^ and Ca^2+^ dynamics. 6. Clinical/translational settings in which INa-L−mediated pro-arrhythmic effects are important include genetically-mediated arrhythmias [18,23,76,79,97–99] ischemia or prolonged free radical insults [100] including diabetic cardiomyopathies [29]. Our computational work does not accurately model these situations. In fact the electrophysiological substrate corresponding to the failing heart is sufficiently different that it requires an additional, separate analysis of the putative pro-arrhythmic effects of I_Na-L_ and related anti-arrhythmic effects/mechanisms of promising drugs such as ranolazine [14,19,23]. In pathophysiological settings [Na^+^]_i_ may increase substantially, although in the human ventricular myocardium this needs to be demonstrated with direct measurement of [Na^+^]_i_.
